# Core-Excited States for Open-Shell Systems in Similarity-Transformed Equation-of-Motion Theory

**DOI:** 10.1021/acs.jctc.4c01181

**Published:** 2025-01-28

**Authors:** Marcos Casanova-Páez, Frank Neese

**Affiliations:** Max-Planck-Institut für Kohlenforschung, Kaiser-Wilhelm-Platz 1, 45470 Mülheim an der Ruhr, Germany

## Abstract

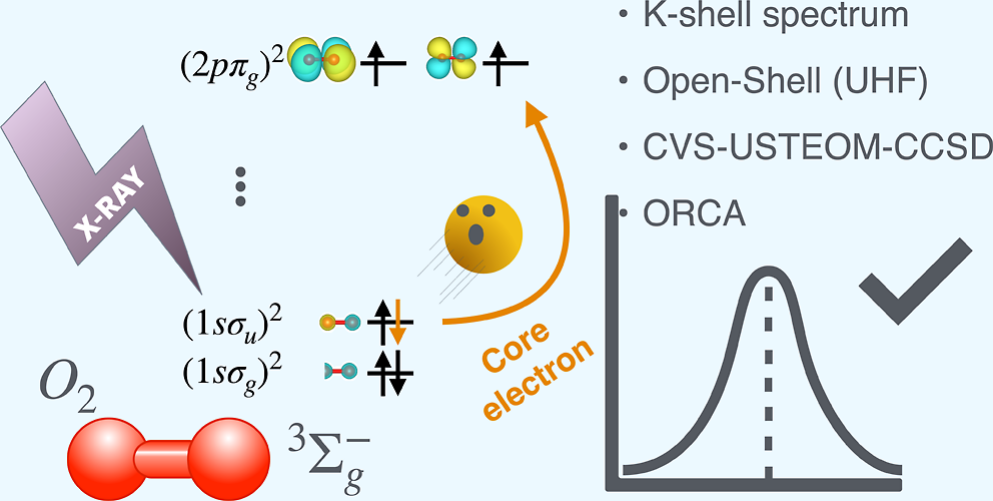

X-ray absorption spectroscopy (XAS) is a powerful method for exploring molecular electronic structure by exciting core electrons into higher unoccupied molecular orbitals. In this study, we present the first integration of the spin-unrestricted similarity-transformed equation-of-motion coupled cluster method (CVS-USTEOM-CCSD) for core-excited and core-ionized states into the ORCA quantum chemistry package. Using the core–valence separation (CVS) approach, we evaluate the accuracy of CVS-USTEOM-CCSD across 13 open-shell organic systems, covering over 20 core excitations with diverse spin multiplicities (doublet, triplet, and quartet). The implementation leverages automated active space selection, incorporating CIS natural orbitals to efficiently capture electronic transitions. We benchmark the predicted K- and L-edge spectra against experimental data, underscoring the accuracy of the CVS-USTEOM-CCSD method for high-precision core excitation studies.

## 1 Introduction

X-ray absorption spectroscopy (XAS), enhanced by modern synchrotron hard- or soft beam sources, is utilized in diverse fields including organic electronics, biological research, among many others.^1−5^ Techniques such as near-edge soft X-ray absorption fine structure (NEXAFS) and X-ray absorption near edge structure (XANES) provide detailed information on chemical structures, molecular orientations, and conduction mechanisms. These methods are crucial in studying phenomena such as tautomerism in DNA bases and resonant intermolecular Coulombic decay (ICD).^6−11^ ICD processes, facilitated by core excitations, have significant potential in targeted cancer radiotherapy by enabling localized energy transfer to neighboring molecules.^12,13^ Core-electron spectroscopy has long been valued for its ability to provide element-specific insights into the electronic structure of materials due to the highly localized nature and distinct energy levels of core orbitals. Recent advancements in X-ray sources, including the advent of tabletop instruments,^14^ have made high-energy X-ray radiation more accessible. These developments have expanded the routine use of XAS to analyze the structure, composition, and electronic distribution of various substances.^15^

A thorough understanding of the underlying theory is essential for accurately analyzing and interpreting experimental results. The use of theoretical simulations has become standard practice for interpreting X-ray spectra obtained from experiments. The literature covers a range of methods for X-ray spectroscopy simulation, including semiempirical models,^16,17^ density functional theory (DFT),^18−28^ and cutting-edge wave function-based techniques.^6,29−41^

Coupled-cluster (CC) theory^42−61^ is a cornerstone method in quantum chemistry renowned for its accuracy and systematic improvement in describing electronic structure. By employing an exponential wave function ansatz and incorporating higher-order excitations, CC methods provide reliable predictions for molecular properties, including ground and excited states. The balance between computational efficiency and accuracy has made CC a valuable tool in various fields. To study excited states, CC theory has been extended through the equation-of-motion (EOM-CC) and linear-response (LR-CC) formalisms.^50,51,55,62−69^ EOM-CC, particularly at the singles and doubles level (EOM-CCSD), excels at determining vertical excitation energies with high accuracy, especially for states dominated by single excitations.^51,62,65,70−86^

While EOM-CCSD deliver accurate excitation energies, their computational cost, scaling with system size as , poses a significant challenge.^87−92^ To mitigate this, approximation techniques such as resolution of identity (RI),^89−92^ Cholesky decomposition (CD),^93−99^ and chain of spheres (COSX)^100,101^ have been developed.

Traditional EOM-CCSD calculations tend to overestimate core excitation energies, largely due to the limitations in accounting for orbital relaxation effects.^31,33,38,41,102^ Hence, applying an energy shift to align calculated spectra with experimental data is a common approach. In our opinion, it is important to note that the “absolute” transition energies in XAS studies are not the most critical aspect of a theoretical X-ray spectroscopic investigation.^35,103^ Deviations between theoretical and experimental values are typically systematic for a given method, basis set, and relativistic treatment. Once shifts for a specific combination have been established through calibration, calculated transition energies are often within experimental resolution.^104,105^ Furthermore, it is important to note that experimental transition energies can vary substantially depending on calibration techniques and therefore comparison between calculated and measured absolute transition energies can be, to some extent, a moving target.^106−108^ Therefore, in our opinion, the focus in theoretical XAS should be on capturing the correct number of initial and final states (e.g., the right number of features), correct relative transition energies, correct relative intensities, and a proper splitting between features, leading to a spectral envelope consistent with the experiment. While absolute energies often receive detailed attention, transition properties such as intensities provide valuable and often critical insights and merit careful analysis.^109^ Last but not least, the calculations also should provide clear insight into the molecular origin of the observed features since this is often of dominant concern to experimentalists which are interested in electronic structure or in spectra/structure relationships.^105^ Thus, ensuring that these aspects are accurately predicted by theoretical methods is essential while having accurate absolute transition energies from a given method is satisfactory but, in our opinion, not crucial.^110,111^

In this study, we focus on the Similarity Transformed EOM-CC (STEOM-CC) method.^112−114^ For optical spectra in the UV/vis region, this method has shown a very favorable combination of high accuracy while being as easy to interpret as a particle-hole approach, such as configuration interaction with single excitations (CIS) or time-dependent density functional theory (TD-DFT).^115−117^ The STEOM-CCSD method involves successive transformations of the Hamiltonian, followed by a diagonalization limited to the space of singly excited determinants. In contrast to the EOM-CCSD method, which requires diagonalization within both singly and doubly excited determinants, STEOM-CC significantly reduces computational cost, particularly when many excitation energies (roots) are calculated. The price to pay for these savings is the introduction of an active space of molecular orbitals which represent the “most readily ionized occupied-” and “most readily populated virtual-” orbitals of the system. Thus, the STEOM-CCSD efficacy hinges on the careful selection of an active space, determined by the preceding IP-EOM-CCSD and EA-EOM-CCSD calculations.^75,118−120,120−122^ Optimal performance requires the amplitudes of both transformation operators to remain relatively small. To achieve this, states with ⟨75% single excitation character in the IP-EOM-CCSD and EA-EOM-CCSD calculations are typically excluded from the treatment as they will lead to unreliable results.

Recent advancements have significantly expanded the capabilities of STEOM-CCSD, encompassing improved efficiency,^80,123,124^ reduced computational demands,^79,124^ and broader applicability to various molecular systems, including open-shell molecules,^124,125^ vibrational motion,^126^ and spin–orbit coupling.^127^ This method excels in providing accurate predictions for different types of excited states, such as valence, Rydberg, and charge-transfer excitations. Its computational efficiency makes it suitable for studying systems requiring the calculation of numerous excited states, like those encountered in XAS. To address the challenges of applying STEOM-CCSD to core-level excitations, we built upon the active space selection scheme originally developed by Dutta and co-workers in 2017.^80^ This scheme was further modified for core-valence separation in closed-shell systems by Ranga and Dutta in 2021.^38^ In this work, we extend this approach for core-valence separation in open-shell molecules.

We recently reimplemented IP-EOM-CCSD, EA-EOM-CCSD, and STEOM-CCSD methods for open-shell systems within the ORCA 6.0 quantum chemistry program.^128,129^ The accuracy of STEOM-CCSD for open-shell organic radicals and transition metal (TM) complexes has been assessed against higher-level methods, including CC3^130,131^ and EOM-CCSDT.^53^ Our reimplementation demonstrates a significant speedup of approximately 2–4 times compared to the previous ORCA 5.0 version thanks to key optimization steps, the most important one being the inclusion of “dressed” integrals quantities which are calculated only once and then reused in subsequent calculations. This approach significantly reduced the computation time and we refer the reader to our recent work in ref (124) for further details.

## 2 Theory

The fundamentals and details of coupled-cluster (CC) theory are widely available in the literature^47^ and in also our recent publication.^124^ Therefore, this work will focus solely on STEOM-CC theory.^112−114^

### 2.1 Similarity Transformed EOM-CC (STEOM-CC) Theory

In STEOM-CC, the similarity-transformed Hamiltonian, ,is subjected to a second similarity transformation, which is expressed with normal ordering ({···}) as
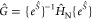
1where the transformation operator *Ŝ* is split into two parts: , with
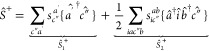
2and
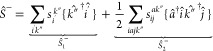
3

Here, the terms with and without dagger denote creation  and annihilation  operators, respectively. The labels *k*″ and *c*″ denote “active” occupied (hole) and virtual (particle) orbitals and the prime symbol refers to inactive orbitals, respectively. The presence of quasiparticle (or *q*-) annihilation operators,  and , in *Ŝ* simplifies the computation of *Ĝ* elements because the different parts of *Ŝ* do not commute. One challenge is that  is generally unknown. However, it has been demonstrated that eq 1 can be rewritten as^132^
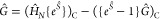
4

For STEOM-CCSD, only the one- and two-body components of Ĝ are needed which simplifies the last equation further by neglecting the contributions arising from the second term, which is known as the renormalization term. The operator *Ĝ* can be expanded in normal order as
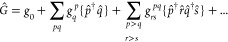
5where the matrix elements of *Ĝ* are derived from the connected contractions of the matrix elements of  and the amplitudes *Ŝ*. Then, *Ĝ* is truncated to include only the two-body elements and the *s* amplitudes in eqs 2 and 3 are obtained by projecting onto an appropriate set of Slater determinants
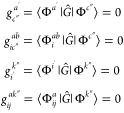
6

It is important to note that the transformation using the operator  preserves the vanishing elements of the original Hamiltonian 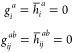
7along with . The matrix representation of the similarity-transformed Hamiltonian, *Ĝ*, is shown in Table 1.

**Table 1:** , , , and  Represent the Sets of Singly, Doubly, Triply, and Quadruply Excited configurations, Respectively^a^

*Ĝ*	|Φ_0_⟩	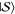	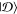	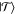	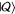
⟨Φ_0_|	Δ*E*	X	X	0	0
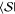	*g*_i_^a^ = 0	X	X	X	0
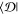	*g*_ij_^ab^ = 0	∼	X	X	X
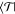	∼	∼	∼	X	X
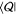	∼	∼	∼	∼	X

a A zero indicates a vanishing element, an *X* signifies a block of significant size, and ∼ denotes small three-particle interactions or ”inactive” two-body operators.

The elimination of the two-particle components of *Ĝ*, specifically , significantly reduces the coupling of an excited determinant to more highly excited determinants. In particular, it removes the primary contribution to the interaction between the singles and doubles configurations in the  block.

The resulting Hamiltonian *Ĝ* is non-Hermitian, and the simplifications are valid only within the lower triangle. By disregarding the negligible elements (∼), we can accurately describe singly excited states through a straightforward diagonalization of *Ĝ* within the subspace of single excitations. This contrasts with EE-EOM-CCSD, where diagonalization of  on both singly and doubly excited state configurations is required to obtain precise excitation energies for singly excited states. The restriction to only singly excitations in the final diagonalization step offers major computational advantages in STEOM-CCSD over EE-EOM-CCSD, particularly when many excited states are to be computed.

In STEOM-CCSD, the focus is exclusively on double *Ŝ* amplitudes due to the impossibility of contracting  to .^112,114,133^ By employing Wick’s theorem, the Hamiltonian transformation is achieved through a two-step process
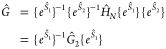
8

Note that *Ĝ* and  must have the same eigenvalue spectrum. Nevertheless, diagonalizing over  is more straightforward making subsequent manipulations more manageable.

For practical purposes, the  amplitudes are determined by directly solving the EA-EOM-CCSD equations, which involve various principal electron affinities. Similarly, the  amplitudes are derived from solving the IP-EOM-CCSD equations for a range of principal ionization energies. Although directly solving eq 6 is possible, it can be challenging to converge.^125,133^ The explicit expressions for the EA and IP s-amplitudes are given by
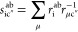
9and
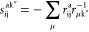
10Here, the matrices  represent the coefficients of the inverse matrix constructed from the principal and active components of the eigenvector *r*_p_^′^(μ) (where μ refers to a specific root).

### 2.2 Automatic Active Space Selection in the CVS Scheme

The STEOM-CCSD method offers an efficient and precise means for analyzing states primarily influenced by single excitations. However, a notable drawback of STEOM-CCSD involves the selection of the active space. To mitigate this issue, Dutta et al. developed a nearly automated approach using valence CIS natural orbitals (NOs) to identify the appropriate active space for STEOM-CCSD.^80^ Building on this, Ranga and Dutta advanced the technique in 2021 for the treatment of core electrons.^38^ In the context of this discussion, capital letters I, J, etc., represent core orbitals, i_v_, j_v_, etc., denote valence occupied orbitals, i, j, etc., refer to arbitrary occupied orbitals, and a, b, etc., refer to unoccupied (virtual) orbitals.

Upon solving the UHF equations, Coulomb and exchange integrals are computed for the subsequent CIS calculation. Specific initial guess vectors are then generated such that they include electrons from core orbitals. Following the resolution of the CIS equations, the state-averaged CIS one-particle reduced matrix for the occupied and virtual blocks is obtained as
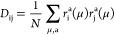
11and
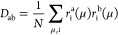
12where *r*_i_^a^ denotes the corresponding CIS eigenvectors associated with the core-excited state μ, and *N* represents the total number of states. Then, the state-averaged CIS natural orbitals (CIS-NOs) are derived by diagonalizing the reduced density matrix blocks for occupied (*D*_ij_) and virtual (*D*_ab_) orbitals. Occupied and virtual orbitals up to a certain threshold are considered as active orbitals. In ORCA, these thresholds are controlled by the keywords “IPTHRESH” and “EATHRESH”, respectively, with default values set to 0.001 after careful testing.^80^

Next, the Fock Matrix is also transformed into the NOs basis
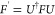
13and the active and inactive blocks of the occupied (o) and virtual (v) subspaces are then diagonalized separately, resulting in a block-diagonal modified Fock matrix
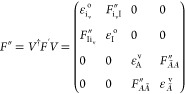
14In the matrix described above, ε denotes the diagonal blocks of the Fock matrix, while , , , and  represent the nonzero off-diagonal blocks of the Fock matrix. Here, *A* and *Ã* denote active and inactive virtual orbitals, respectively.

Finally, the Hartree–Fock atomic orbital (AO) to molecular orbital (MO) coefficients are updated as^38,80^
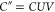
15where *C*″ is the transformation matrix from AO to CIS-NOs basis. The AO integrals and final CIS vectors are transformed to the CIS-NO basis and stored as an initial guess for the subsequent STEOM-CCSD calculation.

Due to the differing structures of the Fock matrices, the Hamiltonian in CVS-IP-EOM-CCSD will exhibit variations depending on whether a canonical or CIS-NOs basis is used. In the canonical MO basis, the highest occupied molecular orbitals are valence orbitals . Conversely, in the CIS-NOs basis within the CVS approximation, the highest occupied molecular orbitals correspond to core orbitals (ε_I_^o^). Therefore, the use of CIS-NOs, which is specific to STEOM-CCSD in the CVS approximation, represents the main difference compared to other CVS approximations, which would be equivalent to canonical MOs.^134^ For additional details and analysis, readers are advised to consult refs (80) and (38).

### 2.3 Calculation of Properties in STEOM-CC

The calculation of oscillator strength and other related properties in STEOM-CCSD is thoroughly described by Ghosh and co-workers in ref (135); here, we provide only a summary. The calculation of oscillator strength requires the transition dipole moment *T*_k_ given by^134,135^
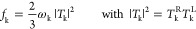
16where ω_k_ represents the vertical excitation energy for state “k”, and R/L refers to the right-/left-hand solutions. If *D*_q_^p^ = ⟨p|**r**|q⟩ is the dipole integral, then the transition dipoles in the length gauge approximation take the form
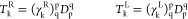
17here, γ_q_^p^ is the one-electron reduced density matrix (1-RDM), which is determined as
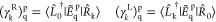
18where  represents the right-hand eigenvector,  the left-hand eigenvector, and  is a shorthand notation for terms involving the ground state  and second transformation  operators.

## 3 Computational Details

All computations were performed using a development version of the ORCA 6.0.1 quantum chemistry software package.^128,129^ The molecular structures analyzed in this study were taken from the work of Zhao and co-workers^40^ and optimized at the unrestricted second-order Møller–Plesset perturbation theory (UMP2)^136^ level using the def2-TZVPP AO basis set,^137^ combined with the resolution-of-the-identity (RI) approximation and its respective auxiliary basis set.^138^

For single-point calculations, the *aug*-cc-pVTZ-DK AO basis set^139−142^ was employed alongside the automatically generated “AutoAux” fitting basis set.^143^ Scalar relativistic effects were incorporated using the second-order Douglas-Kroll-Hess Hamiltonian (DKH2).^144−146^

The automatic space selection scheme was employed for the STEOM-CCSD calculation using the default thresholds, as implemented in ORCA 6.0. We note that the present work does not use the “frozen core” (fc) approximation, meaning that the ground-state CCSD equations are solved by considering all electrons, unlike other implementations commonly referred to as fc-CVS methods.^147^ A full width at half-maximum (fwhm) of 1.0 eV was used for all calculated spectra in this work unless stated otherwise. The ionization potential thresholds were determined by analyzing the results of the preceding CVS-IP-EOM-CCSD calculation.

In 2014, Wenzel and co-workers introduced an open-shell CVS formulation of the extended algebraic diagrammatic construction scheme for the polarization propagator, the CVS-UADC(2)-x method, and applied it to some of the systems studied in this work.^39^ Consequently, we compare our CVS-USTEOM-CCSD results to those reported in ref (39). It should be noted, however, that their calculations utilized the 6-311++G** AO basis set^148,149^ and did not incorporate relativistic effects. We denote these results as CVS-USTEOM-CCSD-x to indicate that the calculation conditions align with those of the ADC(2)-x method and the results can be found in the Supporting Information.

### 3.1 New MultiCore Feature for CVS-USTEOM-CCSD

One limitation of calculating core excitation energies, in the closed-shell STEOM implementation in ORCA, is the restriction to accessing only one core orbital at a time, which necessitates recalculating the computationally expensive ground-state CCSD equation for each core orbital of interest. To address this issue, the next release of ORCA will allow users to define multiple core orbitals in a single CVS-USTEOM-CCSD calculation by introducing two new keywords: “multicoreorb” and “coreorb”. To help clarify their usage, we included input examples with corresponding CIS outputs (representing the CVS-USTEOM-CCSD initial guess vectors) in the Supporting Information. This feature will also be implemented in the closed-shell version for the upcoming ORCA release.

## 4 X-ray Absorption Spectra in Open-Shell Organic Molecules

In this work, the accuracy of CVS-USTEOM-CCSD for a series of open-shell, organic molecules is evaluated, including the triplet dioxygen molecule (O_2_), allyl radical (C_3_H_5_), methyl radical (CH_3_), hydroxyl radical (OH), dinitrogen cation (N_2_^+^), nitrosyl radical (NO), nitrogen dioxide radical (NO_2_), quartet and doublet states of the amidinium ion (NH^+^), triplet amino cation (NH_2_^+^), ammonium cation (NH_3_^+^), carbon monoxide cation (CO^+^), hydroperoxyl radical (OOH), and the phenothiazine cation radical (PTZ^+^), encompassing overall more than 20 core excitations.

The studied radicals and ions include a variety of significant species. The methyl radical (CH_3_) is a fundamental intermediate in organic reactions and combustion processes.^150^ The hydroxyl radical (OH), prevalent in the Earth’s atmosphere, plays a crucial role in oxidation and combustion reactions. The nitrosyl radical (NO) is involved in atmospheric chemistry and biological signaling.^151^ The carbon monoxide cation (CO^+^) is important in astrochemistry and ion–molecule studies. The amidinium ion (NH^+^) is relevant in organic synthesis and radical chemistry. The triplet amino ion radical (NH_2_^+^) is studied in mass spectrometry and plasma chemistry. The ammonium ion (NH_3_^+^) is a common cation in both organic and biological systems. The hydroperoxyl radical (OOH) is significant in atmospheric chemistry and oxidation reactions.^150^ The triplet dioxygen diradical (O_2_) is vital for respiration and combustion,^152,153^ while nitrogen dioxide (NO_2_) is a key pollutant involved in smog and acid rain formation.^154^ The allyl radical (C_3_H_5_) plays a significant role in both hydrocarbon formation and interstellar chemistry. Phenothiazines are bioactive molecules with pharmacological significance, known for their charge transfer properties, radical cation formation, and use in treating psychotic disorders.^155,156^ Collectively, these species cover a range of core excitations relevant to understanding their chemical and environmental roles.

### 4.1 Basis Sets, Relativistic Effects, and Energy Shifts

**Figure 1:**
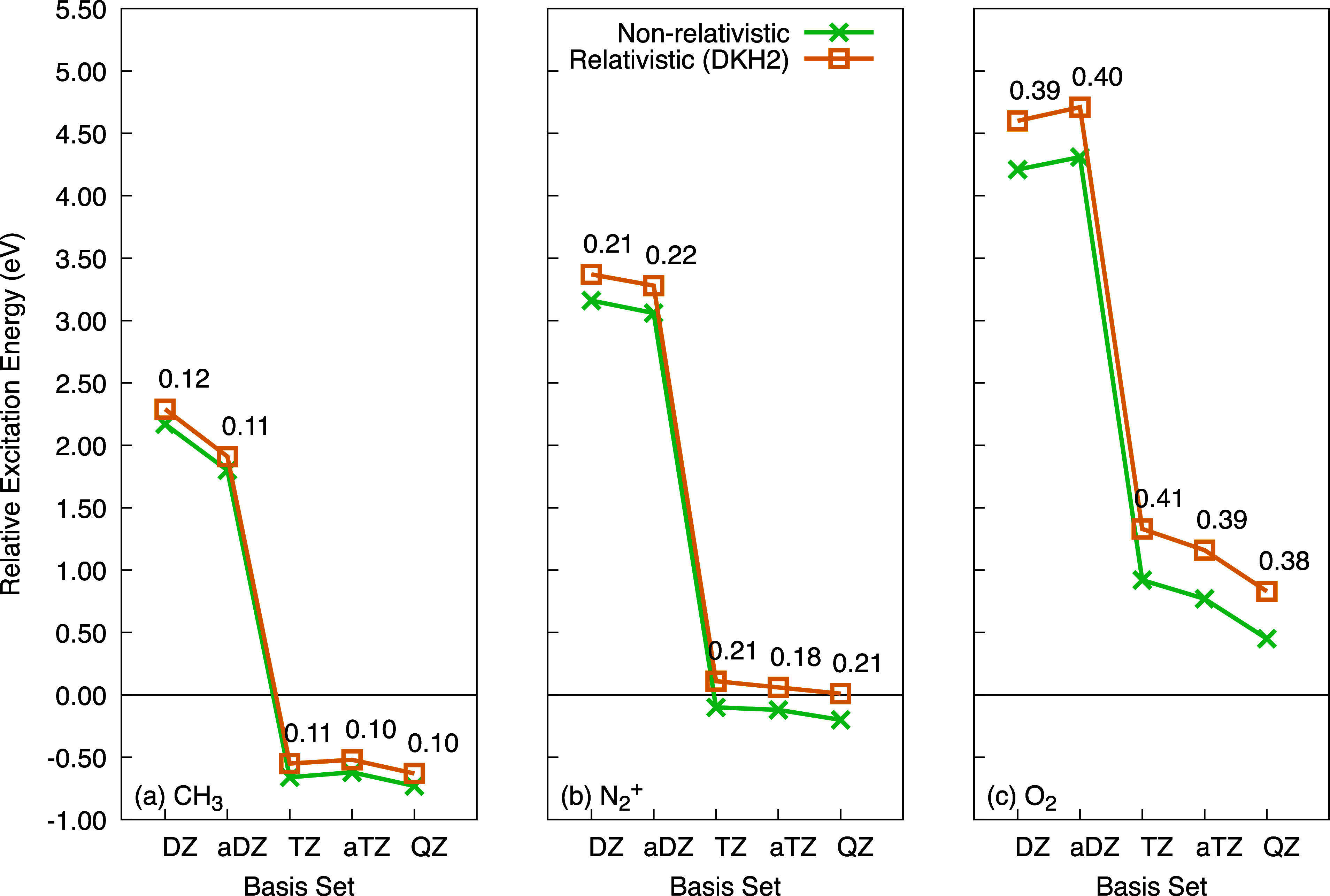
Relative excitation energies for the 1s excitation in the (a) CH_3_ radical, (b) N_2_^+^ ion, and (c) triplet O_2_ molecule, compared to experimental values, using various AO basis sets with and without relativistic corrections. The experimental values are shifted to match 0 eV. The numbers represent the energy differences between relativistic and nonrelativistic calculations.

In Figure 1, we show the convergence of relative excitation energies for the 1s excitation in the CH_3_ radical, N_2_^+^ ion, and triplet O_2_ molecule, compared to their respective experimental values. These calculations use various AO basis sets, ranging from cc-pVDZ (DZ) to cc-pVQZ (QZ), with and without the DKH2 relativistic corrections. Note that we used the appropriate AO basis set when applying the DKH2 correction (DZ-DK, TZ-DK, etc.). We also included the augmented versions, namely aug-cc-pVDZ (aDZ) and aug-cc-pVTZ (aTZ), in our study.

First, we observe that the relativistic corrections closely follow the nonrelativistic relative energies. This indicates that, for a given element, the shifts in absolute transition energies when moving from a nonrelativistic to a scalar relativistic theory remain highly consistent across a series of molecules. Second, the relativistic shifts, ranging between 0.1 and 0.4 eV, are largely independent of the choice of AO basis set. For example, the shifts are 0.11 eV for Carbon and 0.41 eV for Oxygen.

Regarding the effect of enlarging the basis set, the inclusion of additional diffuse functions has minimal impact on excitation energies, except for the DZ AO basis set in the case of Carbon, where the change is more noticeable. In general, excitation energies appear to be largely at the triple-ζ level and the absolute energy shift necessary to align with the experimental value decreases as we increase the size of the basis set. These results allow us to determine an empirical shift (ω_shift_) for each element, which will be applied to all calculated spectra in subsequent analyses (Table 2). These shifts are based on calculations using the *aug*-cc-pVTZ-DK basis set. If smaller basis sets are employed in a particular study, the shifts would need to be adjusted accordingly. However, for larger basis sets, the shifts are expected to remain valid (The remaining data for all the basis sets can be found in the Supporting Information).

**Table 2:** Theoretical Transition Energies (ω) and Energy Shifts (ω_shift_) Computed at the CVS-USTEOM-CCSD Level for the Maxima of the Energetically Lowest K-Edge Absorption Peak for the CH_3_ Radical, N_2_^+^ Ion, and Triplet O_2_ Molecule

system	Exp.^a^	ω	ω_shift_ (Exp.-STEOM)
carbon	281.35	280.73 (280.83)	0.62 (0.52)
nitrogen	394.3	394.18 (394.36)	0.12 (−0.06)
oxygen	530.8	531.57 (531.96)	–0.77 (−1.16)

a Experimental values for CH_3_ are taken from ref (157), N_2_^+^ from ref (158), and triplet O_2_ from refs (159−161). All energies are reported in eV, with DKH2-corrected values shown in parentheses. Calculations were performed using the *aug*-cc-pVTZ-DK basis set.

For basis sets that have already converged, such as TZ or QZ, the absolute energy shift required to align with the experimental values decreases with the atomic number and even becomes negative for oxygen. Therefore, the energy shifts applied throughout this work are +0.52 eV for carbon, −0.06 eV for nitrogen, and −1.16 eV for oxygen. We note that at the TZ basis set without relativistic effects, the shift of −1.16 eV obtained in this work is considerably larger than the previously reported value of −0.71 eV by Chantzis and co-workers^104^ using the CASCI^162,163^/NEVPT2^164,165^ method.

### 4.2 Dioxygen Molecule

**Figure 2:**
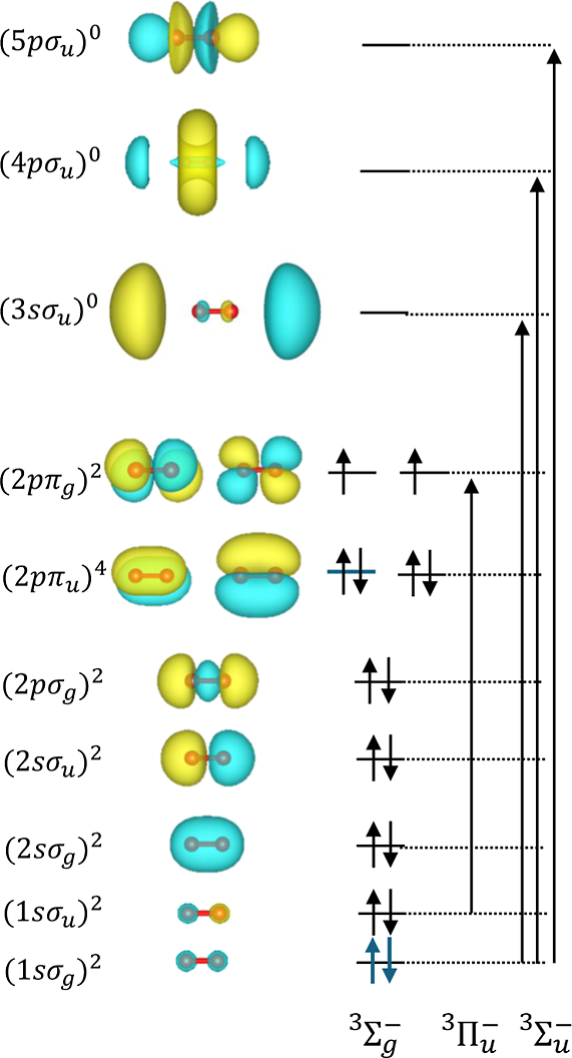
Molecular orbital (MO) diagram for the oxygen diradical (O_2_). The diagram includes the main transitions arising from single electron core excitations, indicated by black vertical arrows. Only dipole-allowed transitions, consistent with the selection rules, are depicted, and the corresponding symmetry of each excitation is also indicated.

The dioxygen molecule belongs to the *D*_∞*h*_ symmetry group, with a ^3^Σ_g_^–^ ground-state symmetry, as shown in the orbital MO diagram in Figure 2. The experimental oxygen K-edge 1s spectrum shows three prominent peaks below the ionization threshold.^159−161^ The first peak, located at 530.8 eV, is attributed to the promotion of an oxygen 1s σ_u_ electron to the lowest unoccupied molecular orbital (LUMO), corresponding to the 2p π_g_ orbital. This results in the configuration (1s σ_u_)^1^ → (2p π_u_)^4^(2p π_g_)^3^, which gives rise to a ^3^Π state. The second and third peaks, with maxima approximately at 539.2 and 541.9 eV, are associated with excitations of the 1s σ_g_ electron to the 3s, 3p, and higher-energy Rydberg levels.

**Figure 3:**
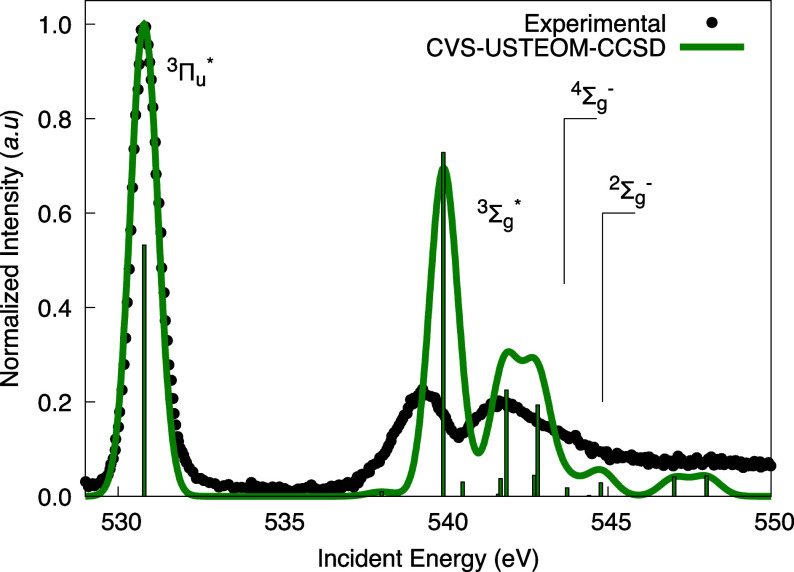
Experimental K-shell spectrum of the dioxygen molecule 1s electrons is compared to the CVS-USTEOM-CCSD calculations. The intensity was normalized to the first peak, and the computed energies were shifted by −1.16 eV. The lines marked ^4^Σ_g_^–^ (543.66 eV) and ^2^Σ_g_^–^ (544.83 eV) indicate the energy positions of the ionization thresholds. Experimental values taken from refs (159−161)

In Figure 3, we present a comparison of the experimental spectra with our corrected theoretical results. The primary features observed in the experimental data are well captured in our calculations. According to our calculations, the ^3^Π_u_ state exhibits the highest intensity in the spectra, while the higher-energy Rydberg states are located beyond 538 eV. The second peak arises from core electron excitation into the (5s σ_u_) orbital, occurring at approximately 539.36 eV. The final peak is a result of a combination of two excited states, with core electrons promoted to the (3p π_u_) and (3s σ_u_) orbitals, with maxima located at 541.89 and 542.84 eV. In general, the triplet O_2_ spectrum is well reproduced by our CVS-USTEOM-CCSD method.

### 4.3 Allyl Radical

**Figure 4:**
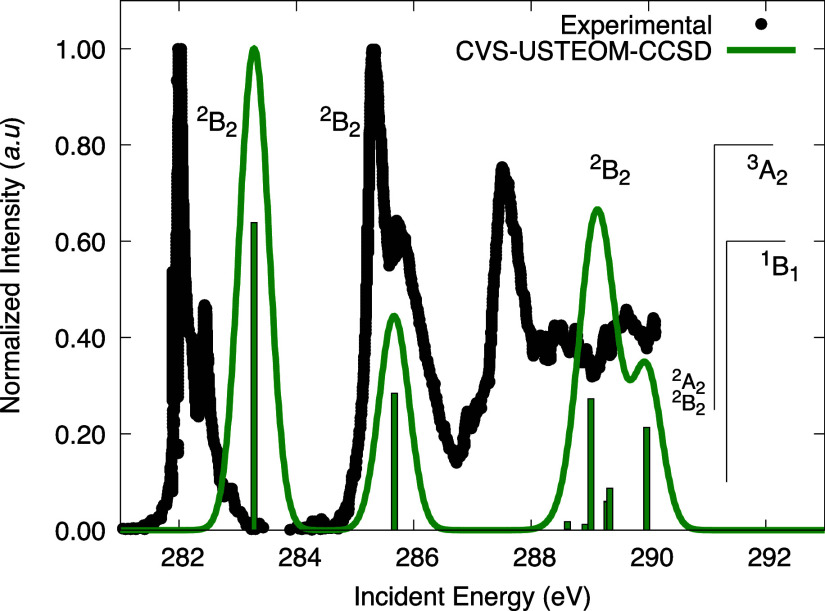
Experimental K-shell spectrum of the allyl radical C K-edge 1s electrons is compared to our CVS-USTEOM-CCSD calculations. The intensity was normalized to the first peak, and the computed energies were shifted by +0.52 eV with a fwhm of 0.6 eV. The lines marked ^3^*A*_2_ (291.12 eV) and ^1^*B*_1_ (291.33 eV) indicate the energy positions of the ionization thresholds. Experimental values taken from ref (166)

Recent experimental investigations by Alagia et al. yielded the first spectrum of the allyl radical (C_3_H_5_, ground state ^2^A_2_), highlighting distinct features associated with its core excitation transitions.^166^ The experimental XAS, depicted in Figure 4, reveals prominent peaks at 282, 285.3, and 287.5 eV, alongside two additional features at 282.5 and 285.7 eV in the low-energy region, corresponding to the first two excitations. These low-energy features exhibit vibrational fine structure with sharp experimental details.

The theoretical X-ray absorption spectrum for the allyl radical, computed using the CVS-USTEOM-CCSD approach, demonstrates reasonable qualitative agreement with the experimental results. The initial peak, situated near 282 eV, corresponds to a transition involving the 1s orbital of the terminal carbon (Ct) to the singly occupied molecular orbital (SOMO). This peak is the most intense in the calculated spectrum, matching the experimental observation. The second prominent experimental peak is predicted as a transition from the central carbon (C_c_) to the LUMO (2-*B*_2_), with a calculated energy of 285.44 eV, although its predicted intensity is somewhat lower than that observed experimentally. The third peak is also attributed to a C_c_ → LUMO (2-*B*_2_) transition, calculated at 288.03 eV, with an intensity that aligns closely with the experimental measurement.

Notably, the features at 282.5 and 285.7 eV, observed experimentally, are absent in the predicted spectrum. These features, often referred to as satellites, are typically indicative of resonant double-excitation processes.^167^ Methods like STEOM-CCSD, that are working in a single particle-hole space, can not describe such excitations by means of their construction. In fact, accurately treating such satellite states would requite the inclusion of (at least) triple excitations. For example, the C_c_ → 2-*B*_2_ excitation leads to two distinct states, 2^2^*B*_2_ and 4^2^*B*_2_, arising from different pathways through the spin-branching diagram. Such excitations can also not be described correctly in a particle-hole picture since the trip-doublet component (a triplet excitation from the core to the virtual space combined with a spin-flip in the SOMO), involves a doubly excited determinant.

### 4.4 Methyl and Hydroxyl Radicals

Methyl radicals (CH_3_) were generated by Alagia and co-workers in the gas phase through rapid thermal decomposition of azomethane within a supersonic helium carrier gas.^157^ The resulting absorption spectrum revealed a vibrational band structure comprising four well-defined peaks in the energy region of 281–283 eV. These spectral features were ascribed to a dominant electronic excitation at 281.35 eV. The core-excited energy calculated with CVS-USTEOM-CCSD was analyzed in the previous Section 4.1 (Table 2), where we determined the energy shift for the carbon atom to align with the experimental value.

The hydroxyl radical (OH) is generated in the gas phase through a reaction involving hydrogen atoms and nitrogen dioxide^168^
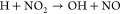


This reaction efficiently produces the OH radical, which is a key species in atmospheric chemistry and combustion processes. The theoretical results for the OH radical are summarized in Table 3.

**Table 3:** Corrected Excitation Energy (ω_corr_ [eV]), Oscillator Strength (*f*_osc_), Spin Expectation Value (⟨*S*^2^⟩), and Symmetry Computed at the CVS-USTEOM-CCSD Level for the CH_3_ and OH Radicals

system	ω_corr_	*f*_osc_	⟨*S*^2^⟩	Sym	Exp.^a^
					
^2^*B*_2_ ground-state					
1s C core					
CH_3_	281.35	0.055	0.82	*B*_2_	281.35
^2^*B*_1_ ground-state					
OH	524.45	0.063	0.79	*A*_1_	525.8

a Experimental values for CH_3_ are taken from ref (157), and for OH from ref (168).

The computed ⟨*S*^2^⟩ value of 0.79 suggests negligible spin contamination in the excited state. The experimental absorption spectrum exhibits a vibrationally resolved peak structure spanning the energy range of 525.0–527.5 eV, corresponding to a single optically active electronic transition with an experimental excitation energy of 525.8 eV. After applying the correction, the theoretical excitation energy deviates by −1.35 eV from the experimental value.

### 4.5 Radicals Involving Nitrogen

#### 4.5.1 N_2_^+^ Radical

The diatomic nitrogen cation (N_2_^+^) belongs to the *D*_∞*h*_ symmetry group and has a ground-state electronic configuration given by
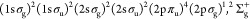


**Figure 5:**
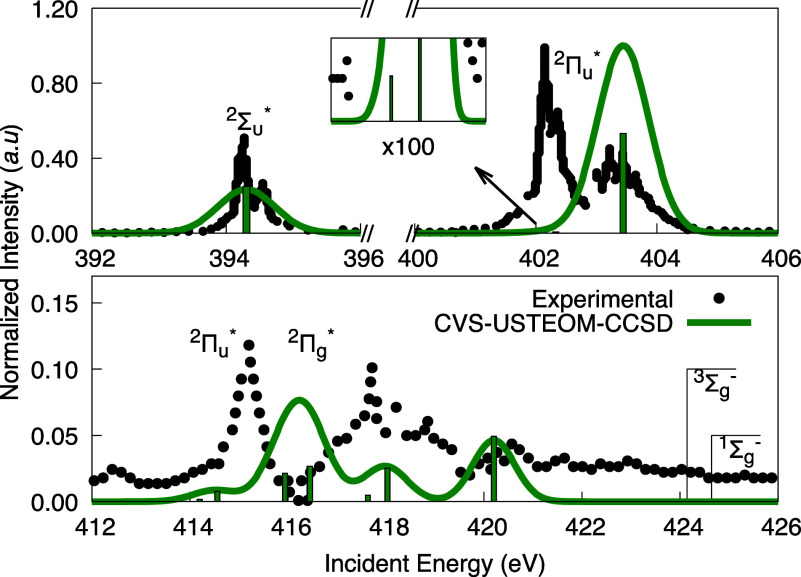
Experimental K-shell spectrum for the N_2_^+^ radical 1s σ electrons is compared with our CVS-USTEOM-CCSD calculations in 3 energy regions. The intensity was normalized to the second peak, and the computed energies were shifted by −0.06 eV. The lines marked ^3^Σ_g_^–^ (424.65 eV) and ^1^Σ_g_^–^ (424.13 eV) indicate the energy positions of the ionization thresholds. Experimental values are taken from ref (158).

Experimental peaks have been observed at 394.3, 402.2, and 403.1 eV.^158^ Our corrected theoretical spectra qualitatively reproduce the experimental data, as depicted in Figure 5.

Within the energy range of 400–405 eV, the experimental spectrum displays two prominent peaks associated with the excitation of (1s σ_u_) core electrons into the (2p π_*g*_) orbitals. In neutral N_2_, the single peak splits into a tripdoublet at 402.2 eV and a singdoublet at 403.1 eV, resulting from spin coupling interactions.^158^ Our corrected calculations predict the first excitation, with reduced intensity (shown in the zoomed box), at 402.30 eV, followed by the second peak, which exhibits the highest intensity, at 403.41 eV. This corresponds to an exchange splitting of 1.11 eV, slightly larger than the experimental value of 0.9 eV.

Additional experimental peaks are observed at 415.2, 417.7, and 418.9 eV, corresponding to excitations from the (1s σ_g_) electron to the 3s σ_u_, 3p π_u_, and other higher-energy Rydberg states. The CVS-USTEOM-CCSD method qualitatively reproduces the overall shape of these lower-intensity peaks, although they are slightly blueshifted relative to the experimental spectrum.

#### 4.5.2 NO_*y*_ (*y* = 1–2) Radicals

The NO radical is primarily characterized by the promotion of (1s σ) core electrons into (2p π_g_) orbitals. Experimentally, the first oxygen K-shell peak is observed at 532.7 eV, attributed to a combination of ^2^Σ^–^, ^2^Δ, and ^2^Σ^+^ symmetries.^161^ The second experimental oxygen K-shell peak appears at 540.2 eV.

The corrected CVS-USTEOM-CCSD calculations predict two states closely aligned with the first experimental peak, at 531.98 and 532.85 eV, as detailed in Table 4. The 2^2^B_1_ state corresponds to an oxygen 1s core → LUMO excitation, while the 2^2^*A*_2_ state arises from a 1s core → SOMO transition. For the second experimental peak, the method predicts two states close in energy at 539.96 and 540.35 eV. The experimental energy gap between the two oxygen K-shell peaks is 7.5 eV. By focusing on the most intense transitions, the CVS-USTEOM-CCSD method identifies the 1^2^*A*_2_ and 2^2^*A*_2_ states, yielding a calculated energy gap of 7.50 eV, in excellent agreement with the experimental result.

**Table 4:** Corrected Excitation Energy (ω_corr_ [eV]), Oscillator Strength (f_osc_), Spin Expectation Value (⟨*S*^2^⟩), and Symmetry Computed at the CVS-USTEOM-CCSD Level for the NO Radical

system	ω_corr_	*f*_osc_	⟨*S*^2^⟩	Sym	Exp.^a^
1^2^*B*_1_ ground-state					
1s O core					
	531.98	0.009	2.54	2^2^*B*_1_	532.7
	532.85	0.061	0.84	1^2^*A*_2_	
NO	539.96	0.000	2.20	3^2^*B*_1_	540.2
	540.35	0.001	1.30	2^2^*A*_2_	
1s N core					
	397.40	0.005	2.68	2^2^*B*_1_	399.7
	399.01	0.101	0.82	1^2^*A*_1_	

a Experimental values for NO are taken from ref (161).

Experimentally, the nitrogen K-shell peak is observed at 399.7 eV. The corrected CVS-USTEOM-CCSD method predicts two low-lying excited states at 397.40 eV (1s core → SOMO) and 399.01 eV (1s core → LUMO). Considering the most intense excitation, the 1^2^*A*_1_ state, the deviation from the experimental value is only −0.69 eV.

In the NO_2_ radical, the experimental oxygen K-shell peaks are observed at 530.3 and 532.4 eV, with an energy gap of 2.1 eV. For the nitrogen K-shell, the experimental peaks appear at 401.0 and 403.3 eV, corresponding to a gap of 2.3 eV.

The CVS-USTEOM-CCSD calculations predict the corrected oxygen 1s excitation energies for the most intense peaks at 530.70 and 533.34 eV, as shown in Table 5, resulting in a calculated gap of 2.64 eV, approximately 0.5 eV larger than the experimental value. Similarly, for the nitrogen 1s excitation energies, the most intense peaks are predicted at 400.39 and 402.57 eV, yielding an energy gap of 2.18 eV, deviating only by −0.12 eV from the experimental value.

**Table 5:** Corrected Excitation Energy (ω_corr_ [eV]), Oscillator Strength (f_osc_), Spin Expectation Value (⟨*S*^2^⟩), and Symmetry Computed at the CVS-USTEOM-CCSD Level for the NO_2_ Radical

system	ω_corr_	*f*_osc_	⟨*S*^2^⟩	sym	Exp^a^
1^2^*A*_1_ ground-state					
1s O core					
	530.70	0.026	0.84	2^2^*A*_1_	530.3
	530.74	0.025	0.84	1^2^*B*_2_	532.4
	532.85	0.007	2.61	1^2^*B*_1_	
	533.34	0.081	0.89	2^2^*A*_1_	
NO_2_	1s N core				
	400.39	0.032	0.80	2^2^*A*_1_	401.0
	400.98	0.000	2.76	3^2^*A*_1_	403.3
	402.57	0.105	0.78	1^2^*B*_1_	

a Experimental values for NO_2_ are taken from ref (169).

#### 4.5.3 NH_*y*_^+^ (*y* = 1 – 3) Radicals

The NH^+^ cation has two closely lying, nearly degenerate ground-state configurations
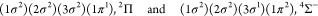
with the corresponding excitation energies summarized in Table 6.

**Table 6:** Corrected Excitation Energy (ω_corr_ [eV]), Oscillator Strength (f_osc_), Spin Expectation Value (⟨*S*^2^⟩), and Symmetry Computed at the CVS-USTEOM-CCSD Level for the NH^+^ Radical

system	ω_corr_	*f*_osc_	⟨*S*^2^⟩	sym	Exp.^a^
1^4^*A*_2_ ground-state					
	394.71	0.045	3.78	2^4^*A*_2_	394.9
	397.59	0.066	3.78	1^4^(*B*_1_ + *B*_2_)	397.8
NH^+^	1^2^*B*_1_ ground-state				
	395.02	0.000	2.76	1^2^*A*_2_	398.8
	398.18	0.155	0.76	2^2^*A*_2_	398.8
	398.45	0.069	0.79	1^2^*A*_1_	399.6

a Experimental values for Nitrogen Hydride were taken from ref (170).

The quartet states arise from excitations of the 1s σ core electron into three different SOMO orbitals: one A_1_ orbital and a *B*_1_ + *B*_2_ orbitals. Experimentally, these states are observed at 394.9 and 397.8 eV, resulting in an energy splitting of 2.9 eV. The CVS-USTEOM-CCSD calculations predict the quartet states at 394.71 and 397.59 eV, with an energy splitting of 2.88 eV and negligible spin contamination, showing excellent agreement with the experimental values.

The doublet states are experimentally observed at 398.8 and 399.6 eV, with an energy gap of 0.8 eV. The CVS-USTEOM-CCSD calculations predict a spin-contaminated excitation at 395.02 eV with negligible absorption intensity, followed by two transitions. The 2^2^*A*_2_ state, located at 398.18 eV, is a superposition of excitations involving the nitrogen 1s core to the SOMO and LUMO orbitals. Meanwhile, the 1^2^*A*_1_ state, located at 398.45 eV, corresponds to a core-to-LUMO excitation. The calculated energy gap between the most intense states is 0.27 eV, which is smaller than the experimental value of 0.8 eV.

**Figure 6:**
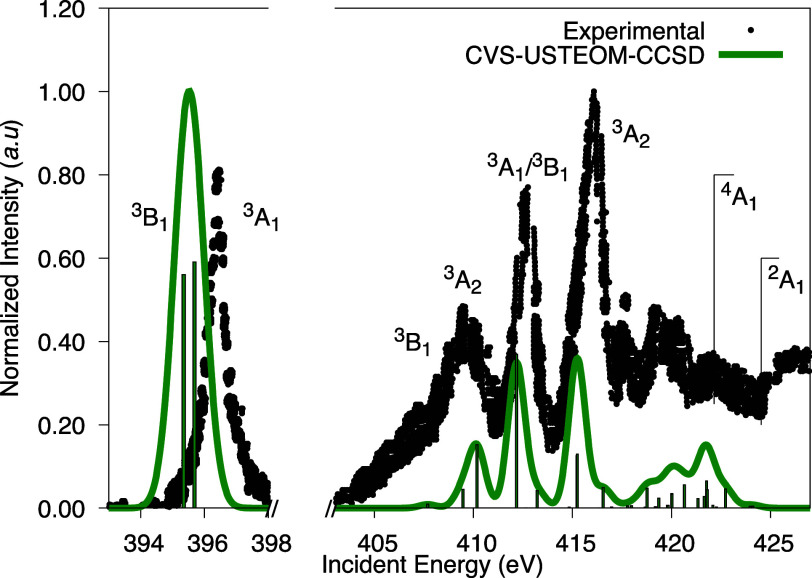
Experimental K-shell spectrum for the NH_2_^+^ radical is compared with our CVS-USTEOM-CCSD calculations. The intensity was normalized to the first peak, and the computed energies were adjusted by −0.06 eV. The lines marked ^4^*A*_1_ (422.52 eV) and ^2^*A*_1_ (424.90 eV) indicate the energy positions of the ionization thresholds. Experimental values are taken from ref (170)

The ground state of NH_2_^+^ exhibits *C*_2*v*_ symmetry with an almost linear geometry, characterized by the ground-state electron configuration , corresponding to the ^3^*B*_1_ state. The CVS-USTEOM-CCSD method qualitatively captures the main features of the experimental spectrum up to 415 eV, as shown in Figure 6.

Due to the nearly planar structure, the calculated energy gap between the first two states is 0.34 eV, whereas these states are experimentally degenerate at 396.4 eV. The next peak in the experimental spectrum appears at 407.2, 10.8 eV higher than the previous two degenerate states. In contrast, the third calculated state, which exhibits a small intensity, is positioned at 407.68 eV, separated by 12.00 eV from the earlier degenerate states.

Beyond this point, the experimental spectrum displays intense peaks with energy gaps of 2.4 eV (3^3^*B*_1_ – 1^3^*A*_2_), 3.1 eV (2^3^*A*_2_ – 2^3^*A*_1_), and 3.4 eV (4^3^*B*_1_ – 2^3^*A*_2_). The calculated energy gaps for these transitions are 1.78, 2.71, and 3.06 eV, respectively.

The calculated energy gaps closely follow the experimental trend but exhibit some small deviations. For instance, the calculated gap of 2.51 eV for the 3^3^*B*_1_ – 1^3^*A*_2_ transition slightly overestimates the experimental value by approximately 0.11 eV. Similarly, the calculated gaps of 1.99 and 3.06 eV for the 2^3^*A*_2_ – 2^3^*A*_1_ and 4^3^*B*_1_ – 2^3^*A*_2_ transitions, respectively, are smaller than the experimental values by 1.11 and 0.34 eV.

**Figure 7:**
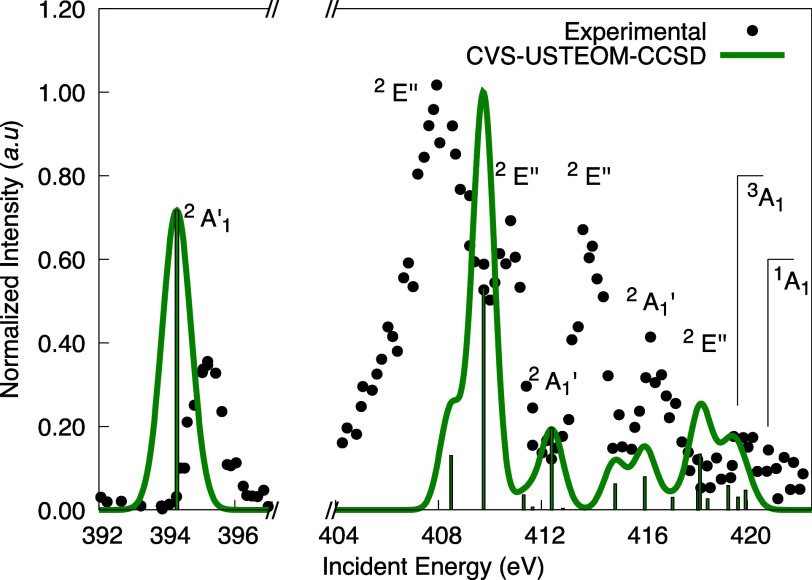
Experimental K-shell spectrum for the NH_3_^+^ radical is compared with our CVS-USTEOM-CCSD calculations. The intensity was normalized to the second peak, and the computed energies were adjusted by −0.06 eV. The lines marked ^3^*A*_1_ (420.57 eV) and ^1^*A*_1_ (421.77 eV) indicate the energy positions of the ionization thresholds. Experimental values are taken from ref (170).

The NH_3_^+^ cation adopts a planar geometry within the *D*_3*h*_ symmetry group and has a ground-state electron configuration of (1*a*_1_^′^)^2^(2*a*_1_^′^)^2^(1*e*^′^)^4^(1*a*_2_^″^)^1^,^2^*A*_2_^″^. The CVS-USTEOM-CCSD method qualitatively reproduces the experimental spectrum, capturing key energy features and transitions as seen in Figure 7.

The first observed experimental transition appears at 395.2 eV and corresponds to the excitation from the 1*a*_1_^′^ orbital to the 1*a*_2_′ SOMO, leading to the ^2^*A*_1_^′^ state. The calculated value for this transition is 394.28 eV, deviating by 0.92 eV from the experimental result.

The subsequent prominent experimental peaks occur at 408.1 and 410.8 eV, corresponding to the two closely spaced ^2^*E*″ states. The calculated excitation energies for these transitions are 408.48 and 409.80 eV, yielding an energy separation of 1.32 eV, smaller compared to the experimental gap of 2.7 eV. Beyond this, the ^2^*A*_1_^′^ state is experimentally located 5.8 eV above the second peak at 408.1 eV. The theoretical prediction places this transition at 412.40 eV, with a gap of 3.91 eV, underestimating the experimental value by 1.89 eV. This larger deviation may reflect the challenges in describing higher-energy transitions within this framework, where states begin to form a continuum and exhibit significant double-excitation character.

### 4.6 Carbon Monoxide Cation and Hydroperoxyl Radicals

The carbon monoxide cation (CO^+^) exhibits a *C*_2*v*_ symmetry, with a ground-state electron configuration of ^2^*A*_1_. The core-excited states for both oxygen and carbon K-shell transitions are well-described by the CVS-USTEOM-CCSD method. For the oxygen K-shell, the first core excitation is predicted at 529.74 eV, in reasonable agreement with the experimental value of 528.5 eV. The second oxygen K-shell transition is computed at 534.14 eV, compared to the experimental value of 533.4 eV. The computed energy gap between these transitions is 4.40 eV, which compares favorably to the experimental gap of 4.9 eV.

For the carbon K-shell transitions, the first excitation energy is 280.46 eV, lower than the experimental value of 282.0 eV. The second and third transitions occur at 287.45 and 289.71 eV, with the second transition showing significant spin contamination (⟨*S*^2^⟩ = 2.04). The predicted energy gap between the first and third transitions is 9.25 eV, which is higher than the experimental value of 7.9 eV.

The hydroperoxyl radical (OOH) belongs to the *C*_*s*_ symmetry group, with a ground-state configuration of ^2^*A*″. For the oxygen K-shell, the CVS-USTEOM-CCSD method predicts the first excitation at 527.48 eV, reasonable close to the experimental value of 528.6 eV. The second excitation occurs at 532.57 eV, with no available experimental value for comparison. Both transitions exhibit low spin contamination, with ⟨*S*^2^⟩ values of 0.79 and 0.85, respectively, as shown in Table 7.

**Table 7:** Corrected Excitation Energy (ω_corr_ [eV])), Oscillator Strength (*f*_osc_), Spin Expectation Value (⟨*S*^2^⟩), and Symmetry Computed at the CVS-USTEOM-CCSD Level for the CO^+^ and OOH Radicals

system	ω_corr_	*f*_osc_	⟨*S*^2^⟩	sym	Exp^a^
1^2^*A*_1_ ground-state					
1s O core					
	529.74	0.008	0.96	2^2^*A*_1_	528.5
CO^+^	534.14	0.032	2.01	1^2^(*B*_1_ + *B*_2_)	533.4
1s C core					
	280.46	0.028	0.98	2^2^*A*_1_	282.0
	287.45	0.013	2.04	1^2^(*B*_1_ + *B*_2_)	289.9
	289.71	0.104	0.98	2^2^(*B*_1_ + *B*_2_)	
1^2^*A*″ ground-state					
OOH	527.48	0.060	0.79	1^2^*A*′	528.6
	532.57	0.044	0.85	2^2^*A*′	

a Experimental values for CO^+^ are taken from ref (171), and for OOH from ref (40).

### 4.7 Phenothiazine Cation Radical

In order to show the potential for the method also for somewhat larger systems, we have studied the phenothiazine cation radical (PTZ^+^). The calculations were performed using an optimized geometry obtained at the CVS-USTEOM-CCSD/cc-pVDZ-DK//B3LYP-D3BJ/def2-TZVPP level of theory.^172,173^ PTZ^+^ belongs to the *C*_*s*_ point group with a ^2^*A*″ ground-state symmetry, and its electronic configuration is summarized as
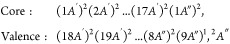


**Figure 8:**
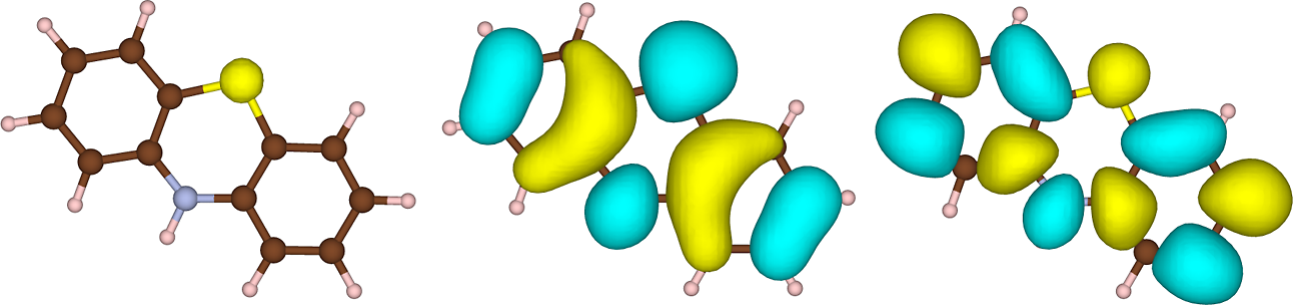
Orbitals in PTZ^+^ relevant to core excitations: sulfur 1s core (left), SOMO (middle), and LUMO (right). Carbon atoms are shown in brown, nitrogen in silver, and sulfur in yellow.

Here, the (1*A*′) and (2*A*′) orbitals correspond to sulfur 1s and nitrogen 2s core orbitals, respectively, followed by 12 consecutive carbon core orbitals, (*XA*′), where *X* = 3 – 14. The primary orbitals involved in core excitations are illustrated in Figure 8.

For PTZ^+^, we employed the cc-pVDZ-DK AO basis set, comprising a total of 346 basis functions. Here, we first compare the excitation energies and then analyze the computational details.

The calculations were carried out on a dedicated node equipped with an AMD EPYC 75F3 32-Core processor. We employed the cc-pVDZ-DK AO basis set, targeted 20 roots, and utilized 8 cores with 64,000 MB/core memory. The number of roots determined by the automatic active space selection are 1 for each IP-EOM calculations (1s core orbital), 10 and 11 roots for each EA-EOM calculation, respectively.

**Figure 9:**
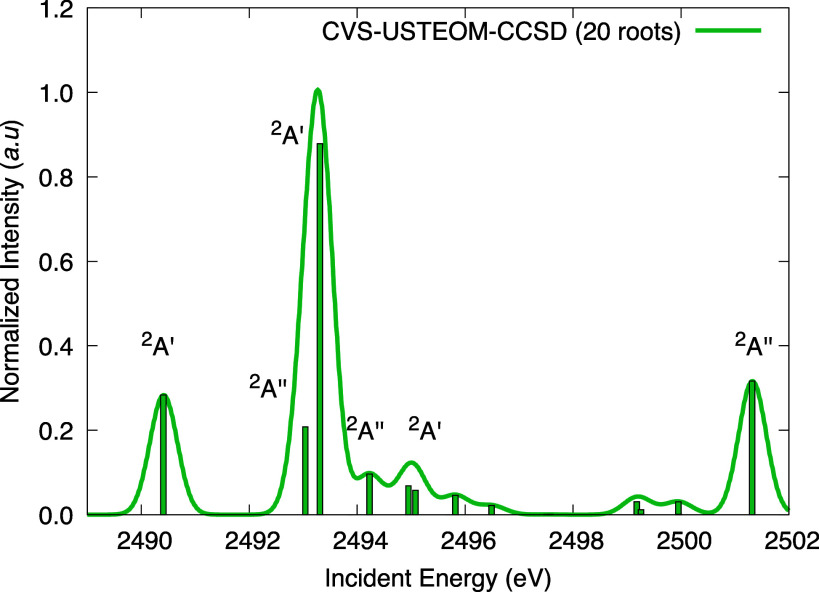
(BT-)CVS-USTEOM-CCSD spectra for sulfur 1s core excitations in PTZ^+^. Intensities are normalized to the highest peak.

In Figure 9, we present the calculated spectra for PTZ^+^ for the 20 requested roots.

From Table 8, we observe that all the excited states exhibit spin contamination, with ⟨*S*^2^⟩ deviations ranging from 1.75 to 2.59 from the ideal value of 0.75. This may be expected by the discussion above that clarifies that in a spin unrestricted approach based on an open-shell ground state, one has to accept certain shortcomings in the description of the spin-couplings of the excited states, in particular if the excitation space only consists of single particle-hole pairs.

**Table 8:** Excitation Energies (ω [eV])), Oscillator Strength (*f*_osc_), Spin Expectation Value (⟨*S*^2^⟩), and Symmetry Computed at the CVS-USTEOM-CCSD Level for the PTZ^+^ Radical^a^

method	ω	*f*_osc_	⟨*S*^2^⟩	sym
1^2^*A*″ ground-state				
USTEOM	2490.41	0.002	1.80	1^2^*A*′
	2493.03	0.001	3.34	2^2^*A*″
	2493.31	0.005	2.05	2^2^*A*′
	2501.32	0.002	1.92	3^2^*A*″

a ⟨*S*^2^⟩ values and symmetry information are currently only available for canonical UHF-EOM calculations.

From the breakdown of the timings in Table 9, one observes that the calculation of the EA roots as well as the determination of the IP roots are the most expensive steps in the calculation while the calculation of the dressed integrals required for the solution of the IP/EA equations as well as the final diagonalization of the singles–singles-block are relatively cheap.

**Table 9:** Computational Timings and Memory Usage for Different Calculations at the CVS-USTEOM-CCSD Level for the PTZ^+^ Radical^a^

Calculation	time
CCSD (16 iterations)	4 h 34 m
per iteration	17 m
integral transformation	1 m
EOM	7 h 56 m
IP-EOM dressing	21 m
EA-EOM dressing	22 m
IP-EOM (37 iterations)	2 h 57 m
EA-EOM (14 iterations)	4 h 7 m
STEOM-CCSD (16 iterations)	6 m
	Memory Usage
maximum memory used	44.3 GB
memory for IP-EOM	10.5 GB
memory for EA-EOM	38.5 GB

a The number of iterations in IP/EA-EOM and STEOM-CCSD is averaged for all the calculated states.

There are two computationally demanding terms: the well-known 4-external (virtual) orbitals, which arise not only in the ground-state CCSD calculation but also in the EA-EOM calculation when solving the doubles–doubles block of the Hamiltonian. Slightly less demanding is the 3-external orbitals term in the EA-EOM calculation, which is mitigated by being precalculated during the dressing step. The overall maximum memory required for this calculation is approximately 44 GB, while the EA-EOM calculation demands slightly less at 39 GB. In contrast, the IP-EOM calculation is significantly less demanding, requiring only 11 GB of memory.

## 5 Conclusions and Future Work

The CVS-USTEOM-CCSD method has demonstrated reasonable high accuracy and reliability across a diverse range of molecular systems, effectively capturing key features in X-ray absorption spectra and aligning closely with experimental data. A detailed analysis of basis set, relativistic effects, and energy shifts were presented for a set of three molecules which was systematically applied in subsequent calculations. Energies were found to converge with the *aug*-cc-pVTZ-DK AO basis set, independent of relativistic effects, which was employed in this work. The significant separation between core and valence orbitals justifies the core–valence separation approximation, greatly reducing the size of the CIS, IP-EOM-CCSD, and STEOM-CCSD spaces, enabling efficient computations. The implementation supports excitation energies, oscillator strengths, and multicore calculations.

CVS-USTEOM-CCSD delivers reliable spectra for most systems studied, with computed relative energies closely matching experimental values after applying consistent atomic energy shifts, typically deviating by < 1 eV. However, reproducing the experimental intensities remains somewhat challenging. In addition to technical factors in the calculations, such as spin contamination and significant double-excitation character, we believe that in the real systems there is a multitude of mechanisms that redistribute the intensity among the available excited states. Such mechanisms, for instance the spin-orbit of Herzberg–Teller coupling, are not taken into account in out present implementation.

The main limitation of the method has nothing to do with the physics incorporated into the STEOM-CCSD Hamiltonian but consists of shortcomings related to spin-coupling. The restriction to a single particle-hole excitation space together with a spin-unrestricted treatment prevents us from forming the proper linearly independent spin-couplings that arise from excitations that lead from the core-orbital(s) to virtual orbitals. This implies that a restricted open-shell treatment, such as the one recently developed in the framework of the ROCIS method,^174^ that can deal with arbitrary spin-couplings in the ground state,^175^ would be an ideal companion to the efficient and compact way in which STEOM-CCSD treats the physics of the excitation process. We are currently investigating possibilities in which such a combination could be achieved.

The allyl radical spectrum highlights a second, related limitation of CVS-USTEOM-CCSD: its inability to describe satellite states, which typically involve significant double-excitation character such as Auger decay processes. Consequently, only one of these peaks can be captured within a single particle-hole picture. Nonetheless, the method still provides a qualitatively accurate spectrum compared to the experimental results.

We also reported results for the phenothiazine cation radical using a smaller AO basis set (cc-pVDZ-DK). Our results show that both energetic and transition properties for more complicated molecules, paving the way for the exploration of larger systems.

In summary, CVS-USTEOM-CCSD is a robust tool for predicting core-excited states across a variety of radicals and cations. The method achieves close alignment with experimental spectra, effectively capturing the primary spectral features and key transitions. With additional diagnostics now available, such as spin expectation values and symmetry information, the method provides valuable tools for interpreting complex spectra. This combination of predictive accuracy and insightful diagnostics underscores the potential of CVS-USTEOM-CCSD as a powerful method for studying core-excited states in diverse molecular systems, while also highlighting areas for further refinement.

We are currently working on implementing the UHF-IP/EA/STEOM-CCSD method in the DLPNO approximation, similar to the implementation already available in ORCA for closed-shell molecules.^176,177^ This development will significantly reduce the computational cost, enabling the exploration of larger molecular systems. It is to be expected that the indicated advancements, namely the combination with local correlation techniques and a more rigorous treatment of excited state spin-coupling, will enhance the accuracy and efficiency of excited-state calculations for open-shell systems and broad the scope of systems that can be studied with high-level, wave function based ab initio methods.

## Supporting Information Available

Raw data associated with each figure and table, in addition to the CVS-USTEOM-CCSD excitation energies, oscillator strengths, and symmetries for the AO basis sets used in this work. We also provide examples of the new feature “multicoreorb” introduced in this work (XLSX)
(PDF)
