# Supplementary material for: Core-Excited States for Open-Shell Systems in Similarity-Transformed Equation-of-Motion Theory
Source: J Chem Theory Comput. 2025 Jan 28;21(3):1306–21. doi: 10.1021/acs.jctc.4c01181 (PMC11823418; doi:10.1021/acs.jctc.4c01181)
Supplement: Supplementary file 2 — ct4c01181_si_002.pdf [file ct4c01181_si_002.pdf]

Supporting Information (I) for: Core-Excited States for Open-Shell Systems in Similarity-Transformed Equation-of-Motion Theory

Marcos Casanova-Páez and Frank Neese**

Max-Planck-Institut für Kohlenforschung, Kaiser-Wilhelm-Platz 1,

45470 Mülheim an der Ruhr, Germany

E-mail: casanova@kofo.mpg.de; neese@kofo.mpg.de

Table of Contents

Section SI. 1: New MultiCore feature for CVS-USTEOM-CCSD 3

Section SI. 2: Core excitations in CVS-UIP-EOM-CCSD 8

Section SI. 3: Geometry optimized coordinates for the molecules used in this work...............11

Section SI. 1: New MultiCore feature for CVS-USTEOM-CCSD

In the forthcoming release of the ORCA computational chemistry software, a significant enhancement will be introduced that allows users to perform simultaneous multiple core excitations within a single CVS-USTEOM-CCSD calculation. This advancement is particularly noteworthy as it obviates the need for repeated computations of the costly ground-state CCSD equations, thereby improving computational efficiency and resource management.

Two new keywords have been incorporated into the ORCA code: “multicoreorb” and “coreorb.” The “multicoreorb” keyword serves to activate the multitask core excitation capability, while the “coreorb” keyword specifies the orbital window to be utilized during the generation of the initial guess for Configuration Interaction Singles (CIS).

$$\begin{aligned} &\text{multicoreorb} \qquad true/false \\ &\text{coreorb}[0] \;=\; i_0,i_1,a_0,a_1 \qquad (\text{Spin Up}) \\ &coreorb[1] \;=\; \bar{i}_0,\bar{i}_1,\bar{a}_0,\bar{a}_1 \qquad (Spin\ Down) \end{aligned}$$

The default values for “coreorb” are all set to -1, indicating that all orbitals are to be used (first occupied MO, HOMO, LUMO, and last unoccupied MO). For instance, in Table S. 1 we present an input for the N_2^+ molecule.

Table S. 1: Input example for the N_2^+ molecule using Multicoreorb and Coreorb[0/1]

```
%mdci
    Nroots                      10
    DoCVS                       True
    CVSEP                       True
    DoCore                      True
    Multicoreorb                True
    Coreorb[0]=                 0,1,-1,-1
    Coreorb[1]=                 0,1,-1,-1
    MaxIter                     100
end
```

This input directly access the first two core electrons from each spin channel (spin up and down), that is, the 1*s* electrons from each N atom, displayed in Table S. 2.

Table S. 2: Selected core orbitals for the CIS initial guess

```
             SPIN UP ORBITALS
  NO   OCC        E(Eh)             E(eV)      Irrep
   0   1.0000    -16.201340     -440.8609    1-Ag
   1   1.0000    -16.197729     -440.7626    1-B1u
             SPIN DOWN ORBITALS
  NO   OCC        E(Eh)             E(eV)      Irrep
   0   1.0000    -16.180328     -440.2891    1-Ag
   1   1.0000    -16.177947     -440.2243    1-B1u
[…]
   5   1.0000     -1.028102       -27.9761    1-B2u
   6   0.0000     -0.523246       -14.2382    3-Ag
```

The default values of -1 for the unoccupied indices indicate that electrons from the LUMO until the last unoccupied MO are to be used in the generation of the CIS initial guess. Then, the final CIS excitation energies will only include excitations from the selected MOs, as seen in Table S. 3.

Table S. 3: UHF CIS results with the corresponding orbital contributions to the excited states.

```
---------------
UHF CIS RESULTS
---------------
IROOT=  1: 14.884326 au   405.023 eV 3266731.9 cm**-1 <S**2>= 0.839046  Sym: B1u
   0b ->  16b    0.044242 (-0.210337)
   1b ->   6b    0.912923 ( 0.955470)
   1b ->  10b    0.017738 (-0.133185)
   1b ->  13b    0.018420 (-0.135721)
```

(continued on next page …)

```
IROOT=  2: 14.888607 au   405.140 eV 3267671.6 cm**-1 <S**2>= 0.839921  Sym: Ag
   0b ->   6b    0.913221 (-0.955626)
   0b ->  10b    0.017563 ( 0.132525)
   0b ->  13b    0.018312 ( 0.135322)
   1b ->  16b    0.044112 ( 0.210028)
```

When core orbitals possess similar energies, both are likely to contribute to excitation processes. However, for orbitals with significant energy differences, the lowest-energy excitation is more likely to happen. For instance, consider the NO molecule and the input provided in Table S. 1

Table S. 4: UHF CIS results for the NO molecule using the input from Table S. 1

```
              SPIN UP ORBITALS
  NO   OCC          E(Eh)            E(eV)    Irrep
   0   1.0000    -20.698590     -563.2373    1-A1
   1   1.0000    -15.729241     -428.0144    2-A1
[…]
              SPIN DOWN ORBITALS
  NO   OCC          E(Eh)            E(eV)    Irrep
   0   1.0000    -20.686881     -562.9186    1-A1
   1   1.0000    -15.697467     -427.1498    2-A1
[…]

---------------
UHF CIS RESULTS
---------------
```

```
IROOT=  1: 14.973200 au   407.441 eV 3286237.6 cm**-1 <S**2>= 2.576438  Sym: A2
   1a ->   8a    0.178253 (-0.422200)
   1a ->  11a    0.034455 ( 0.185621)
   1b ->   7b    0.658996 ( 0.811786)
   1b ->  10b    0.111757 ( 0.334301)
   1b ->  15b    0.010413 ( 0.102046)
IROOT=  2: 15.045207 au   409.401 eV 3302041.3 cm**-1 <S**2>= 0.856996  Sym: A1
   1b ->   8b    0.839133 ( 0.916042)
   1b ->  11b    0.140835 ( 0.375280)
   1b ->  16b    0.014506 (-0.120439)
 […]
(continued on next page …)


IROOT=  9: 15.930824 au   433.500 eV 3496411.7 cm**-1 <S**2>= 0.888665  Sym: B1
   1a ->   9a    0.213464 (-0.462022)
   1a ->  12a    0.413884 (-0.643338)
   1a ->  13a    0.057070 (-0.238894)
   1a ->  14a    0.020240 ( 0.142268)
   1b ->   9b    0.097760 (-0.312665)
   1b ->  12b    0.179885 ( 0.424129)
   1b ->  14b    0.011792 ( 0.108590)
IROOT= 10: 15.954229 au   434.137 eV 3501548.6 cm**-1 <S**2>= 0.808380  Sym: A2
   1a ->   8a    0.070022 ( 0.264618)
   1a ->  11a    0.555002 ( 0.744984)
   1b ->   7b    0.042340 ( 0.205766)
   1b ->  10b    0.321933 (-0.567391)
```

The oxygen 1s core orbital (orbital 0) lies more than 100 eV lower in energy than the nitrogen 1s core orbital (orbital 1). Consequently, the CIS initial guess will exclusively comprise excitations of the type 1a/1b → all virtual MOs. In this scenario, limiting excitations to a reduced manifold of virtual MOs becomes necessary. Consider now the following input given in Table S. 5:

Table S. 5: Input example for the NO molecule after suturing the unoccupied MOs.

```
%mdci
  Nroots                    10
  DoCVS                     True
  CVSEP                     True
  DoCore                    True
  Multicoreorb              True
  Coreorb[0]=               0,1,-1,9
  Coreorb[1]=               0,1,-1,9
  MaxIter                   100
end
```

Herein, the possible excitations that are allowed are of the form 0a/1a → LUMO(a)/9a and 0b/1b → LUMO(b)/9b. Therefore, the CIS initial guess vectors will constrain the possible virtual orbitals and produce the desired results, seen in Table S. 6.

Table S. 6: UHF CIS results for the NO molecule with the saturated unoccupied MOs.

```
---------------
UHF CIS RESULTS
---------------
IROOT=  1: 14.973200 au   407.441 eV 3286237.6 cm**-1 <S**2>= 2.576438  Sym: A2
    1a ->   8a    0.178253 ( 0.422200)
    1a ->  11a    0.034455 (-0.185621)
    1b ->   7b    0.658996 (-0.811786)
    1b ->  10b    0.111757 (-0.334301)
    1b ->  15b    0.010413 (-0.102046)
IROOT=  2: 15.045207 au   409.401 eV 3302041.3 cm**-1 <S**2>= 0.856996  Sym: A1
    1b ->   8b    0.839133 (-0.916042)
    1b ->  11b    0.140835 (-0.375280)
    1b ->  16b    0.014506 ( 0.120439)
[…]
IROOT=  6: 20.034553 au   545.168 eV 4397076.1 cm**-1 <S**2>= 2.292448  Sym: A2
    0a ->   8a    0.818479 ( 0.904699)
    0a ->  16a    0.095700 (-0.309354)
    0b ->   7b    0.064976 ( 0.254905)
    0b ->  15b    0.012795 ( 0.113114)
IROOT=  7: 20.148481 au   548.268 eV 4422080.5 cm**-1 <S**2>= 1.228267  Sym: A2
    0a ->   8a    0.074617 ( 0.273161)
    0b ->   7b    0.831480 (-0.911855)
```

```
   0b ->  15b    0.083164 (-0.288382)
```

Thus, the first half of the excitations are originated from the 1a/1b orbitals, while the latter half arise from the 0a/0b orbitals. Finally, the selection of the active space proceeds as explained in the main manuscript.

Table S. 7: Final active space for the NO molecule with the saturated unoccupied MOs.

```
------------------------------------------
STATE AVERAGED NATURAL ORBITALS FOR ACTIVE SPACE SELECTION (alpha)
------------------------------------------

Solving eigenvalue problem for the occupied space  ... done
Solving eigenvalue problem for the virtual space   ... done
After automatic selection of the active space:
 No of roots active in IP (alpha) calculation:    2
 No of roots active in EA (alpha) calculation:    4

[...]
------------------------------------------
STATE AVERAGED NATURAL ORBITALS FOR ACTIVE SPACE SELECTION (beta)
------------------------------------------

Solving eigenvalue problem for the occupied space  ... done
Solving eigenvalue problem for the virtual space   ... done
After automatic selection of the active space:
 No of roots active in IP (beta) calculation:    2
 No of roots active in EA (beta) calculation:    6
```

Section SI. 2: Core excitations in CVS-UIP-EOM-CCSD

The CVS-IP-EOM-CCSD calculation is automatically executed when CVS-USTEOM-CCSD is requested. The number of roots to be computed is determined from the preceding CIS

calculation, as detailed in the previous section. The structural modifications of the Fock matrices (see main manuscript) are handled internally, requiring no additional input or adjustments from the user.

Table S. 8: Core ionization for the removal of an electron with spin up and down, respectively, for the NO molecule calculated using CVS-USTEOM-CCSD.

-------------------------------	-------------------------------
UHF IP-EOM-CCSD RESULTS (RHS)	UHF IP-EOM-CCSD RESULTS (RHS)
-------------------------------	-------------------------------
IROOT= 1: 15.230585 au 414.445 eV	IROOT= 1: 15.204628 au 413.739 eV
3342727.1 cm**-1 Sym: B2	3337030.2 cm**-1 Sym: B2
Amplitude Excitation	Amplitude Excitation
0.894855 7a -> x	0.901291 6b -> x
0.032417 4a -> x 7a -> 8a	0.032859 2b -> x 6b -> 7b
-0.014674 4a -> x 7a -> 10a	0.016599 2b -> x 6b -> 10b
0.015768 5a -> x 7a -> 12a	0.033839 4b -> x 6b -> 8b
0.167815 7a -> x 2b -> 7b	0.017544 4b -> x 6b -> 11b
0.115008 7a -> x 2b -> 10b	0.172234 6b -> x 4a -> 8a
0.172668 7a -> x 4b -> 8b	-0.109579 6b -> x 4a -> 10a
0.115055 7a -> x 4b -> 11b	0.130461 6b -> x 5a -> 12a
Percentage singles character= 80.08	Percentage singles character= 81.23
IROOT= 2: 20.115526 au 547.371 eV	IROOT= 2: 20.105379 au 547.095 eV
4414847.7 cm**-1 Sym: B2	4412620.7 cm**-1 Sym: B2
Amplitude Excitation	Amplitude Excitation
0.910287 6a -> x	0.911726 5b -> x
-0.026009 4a -> x 6a -> 8a	-0.022336 2b -> x 5b -> 7b
-0.015616 4a -> x 6a -> 10a	-0.022572 4b -> x 5b -> 8b
-0.013827 5a -> x 6a -> 16a	0.014418 4b -> x 5b -> 11b
-0.144713 6a -> x 2b -> 7b	-0.156148 5b -> x 4a -> 8a
0.101191 6a -> x 2b -> 10b	-0.110347 5b -> x 4a -> 10a

-0.149610 6a -> x 4b -> 8b<br>0.103084 6a -> x 4b -> 11b<br>Percentage singles character= 82.86<br><br>IP (Alpha) STATE= 1: percentage singles 80.077<br>IP (Alpha) STATE= 2: percentage singles 82.862<br>Final no active IP roots: 2	-0.109556 5b -> x 5a -> 16a<br>Percentage singles character= 83.12<br><br><br>IP (Beta) STATE= 1: percentage singles 81.233<br>IP (Beta) STATE= 2: percentage singles 83.124<br>Final no active IP roots: 2

Due to the changes in the Fock matrix, the core ionizations are labeled as 7a/6a and 6b/5b, as seen in Table S. 8. However, these two orbitals correspond to the orbitals specified in the input.

Alternatively, if the user wants to calculate only the core ionization energies without performing the STEOM calculations, the input should be adjusted as follows:

Table S. 9: Input and output of the core ionization calculation for the NO molecule with the CVS-IP-EOM-CCSD method.

```
%mdci
 Nroots 5
 DoCVS true
 CVSEP true
 DoCore true
 Dorootwise true
end

Output:
-------------------------------
UHF IP-EOM-CCSD RESULTS (RHS)
-------------------------------

IROOT=  1: 20.115509 au   547.371 eV 4414843.9 cm**-1 Sym: B2
  Amplitude    Excitation
   0.910287     0a -> x
   0.126429     0a -> x     4b ->   7b
```

```
 -0.131438    0a -> x    4b ->  15b
  0.129431    0a -> x    6b ->   8b
 -0.135984    0a -> x    6b ->  16b
Percentage singles character=    82.86

IROOT=  2: 15.230575 au   414.445 eV 3342724.9 cm**-1 Sym: B2
  Amplitude    Excitation
  0.894855     1a -> x
 -0.145079     1a -> x    4b ->   7b
 -0.149130     1a -> x    4b ->  10b
 -0.117509     1a -> x    5b ->  12b
 -0.149144     1a -> x    6b ->   8b
 -0.149691     1a -> x    6b ->  11b
Percentage singles character=    80.08
```

Section SI. 3: Geometry optimized coordinates for the molecules used in this work

```
==>       allyl.xyz   <==
8
C        0.000000   0.000000   0.439479
C        0.000000   1.222300   -0.204919
C        0.000000   -1.222300  -0.204919
H        0.000000   0.000000   1.521627
H        0.000000   2.148655   0.346925
H        0.000000   -2.148655  0.346925
H        0.000000   1.275634   -1.284337
H        0.000000   -1.275634  -1.284337

==>       ch3.xyz    <==
4

C        0.000000   0.000000   0.000000
H        0.537114   0.930309   0.000000
H        0.537114   -0.930309  0.000000
H        -1.074228  -0.000000  0.000000

==>       cop.xyz    <==
2

C        0.000000   0.000000   -0.625286
```

```
O          0.000000    0.000000   0.466616

==>        n2p.xyz    <==
2
N          0.000000    0.000000   0.548961
N          0.000000    0.000000   -0.548961

==>        nh2p.xyz    <==
3

N          0.000000    0.000000   0.064170
H          0.000000    0.993147   -0.200139
H          0.000000    -0.993147  -0.200139



==>        nh3p.xyz    <==
4

N          -0.000000   -0.000000  0.000036
H          0.000000    1.017654   0.000039
H          -0.881314   -0.508827  0.000039
H          0.881314    -0.508827  0.000039

==>        nhp_12.xyz  (S=1/2) <==
2

N          0.000000    0.000000   0.129382
H          0.000000    0.000000   -0.933837

==>        nhp_32.xyz  (S=3/2) <==
2

N          0.000000    0.000000   0.128706
H          0.000000    0.000000   -0.955847

==>        no.xyz     <==
2

N          0.000000    0.000000   0.004908
O          0.000000    0.000000   1.141092

==>        no2.xyz    <==
```

```
3

O          0.000000   1.104808   -0.142814
N          0.000000   0.000000   0.324825
O          0.000000   -1.104808  -0.142814

==>        o2.xyz     <==
2

O          0.000000   0.000000   -0.007734
O          0.000000   0.000000   1.215234

==>        oh.xyz     <==
2

O          0.000000   0.000000   0.001301
H          0.000000   0.000000   0.968399

==>        ooh.xyz    <==
3

O          0.028522   0.680538   0.000000
O          0.028522   -0.624449  0.000000
H          -0.905408  -0.890250  0.000000

==>        ptzp.xyz    <==
23

C          -0.006006  0.000857   0.000107
C          1.368895   0.006357   0.001701
C          2.075666   1.219581   0.001449
C          1.396394   2.418132   -0.000401
C          -0.004359  2.433139   -0.002046
C          -0.716020  1.212028   -0.001793
N          -2.086492  1.184670   -0.003368
C          -2.949682  2.249529   -0.005358
C          -4.333196  2.010659   -0.006790
C          -5.215978  3.064719   -0.008791
C          -4.744769  4.387385   -0.009424
C          -3.390769  4.641577   -0.008041
C          -2.475712  3.580953   -0.005993
S          -0.789439  3.977318   -0.004329
H          -0.552735  -0.933248  0.000299
H          1.907273   -0.930407  0.003158
```

H	3.155924	1.215388	0.002711
H	1.937471	3.354516	-0.000601
H	-2.512250	0.268246	-0.003043
H	-4.693753	0.990194	-0.006299
H	-6.279068	2.871907	-0.009884
H	-5.444773	5.210166	-0.011001
H	-3.024363	5.659085	-0.008524
